# Exploring the Evidence Base on the Association Between Parents’ Adverse and Positive Experiences and the Developmental, Psychological and Behavioral Outcomes of Their Offspring: A Scoping Review

**DOI:** 10.3390/healthcare14152253

**Published:** 2026-07-23

**Authors:** Miriam Riaz Nichol, Christie O’Donnell, Irina Campbell, Carole Murphy, Christopher Graham

**Affiliations:** The Department of Psychological Sciences & Health, University of Strathclyde, Glasgow G1 1XQ, UK; christie.o-donnell@strath.ac.uk (C.O.); irina.campbell@strath.ac.uk (I.C.); carole.murphy.100@strath.ac.uk (C.M.); christopher.graham@strath.ac.uk (C.G.)

**Keywords:** adverse experiences, child outcomes, intergenerational trauma, mental health, parent-child dyads, positive experiences, psychological trauma, resilience and protective factors

## Abstract

**Highlights:**

**What are the main findings?**

- Exposure to adversity across the lifespan can have intergenerational effects, contributing to negative developmental, psychological and behavioral outcomes in offspring.
- The preliminary and ambivalent nature of the current evidence, particularly for positive experiences, warrants further adequately powered examinations that focus on male and non-biological primary caregivers as well as adolescents.

**What are the implications of the main findings?**

- Recognizing that parents’ experiences can contribute to the trajectories of offspring is essential for the development of interventions that not only seek to prevent the intergenerational transmission of adversity but also those that seek to promote resilience.
- The limitations in the evidence base demonstrate that practitioners should heed current findings with caution.

**Abstract:**

Research has demonstrated the intergenerational effects of parents’ adverse and positive experiences. Despite growth in the literature, previous reviews have primarily focused on the intergenerational effects of parents’ childhood experiences. This scoping review aimed to characterize the intergenerational research on the associations between parental adverse and positive experiences across the life course (including childhood and adulthood) and the developmental, psychological and behavioral outcomes of their offspring. Following a search of ten databases, seven articles met the inclusion criteria. The majority of studies were cross-sectional (*n* = 4) and published in the United States (*n* = 6). Female caregivers dominated the data set (84.6%), with the large majority having a sample size above *n* = 1000. All studies reported a significant association between parent adversity and negative offspring outcomes; however, mixed findings were found for the protective properties of parents’ positive experiences. Findings indicate a need for further exploration, underscoring the importance of adequately powered studies inclusive and diverse in nature.

## 1. Introduction

The seminal Adverse Childhood Experiences (ACE) study [1] demonstrated a positive dose–response relationship between childhood maltreatment or household dysfunctions and later health outcomes. Felitti et al.’s [1] study contributed to a paradigm shift in the adversity literature by not only acknowledging the prevalence of childhood maltreatment and household dysfunctions but by emphasizing their cumulative and chronic nature [1,2]. Since then, scholars have proliferated the trauma literature seeking to ascertain the role of childhood maltreatment on life course trajectories [3]. For instance, researchers have demonstrated that early exposure to ACEs is a major risk factor for adverse outcomes, including poor physical health (e.g., diabetes, cancer, cardiovascular diseases) [4,5], poor social functioning (e.g., social isolation, loneliness) [6,7], cognitive impairments (e.g., poorer working, immediate and delayed memory) [8], psychopathology (e.g., depression, anxiety, suicide ideation, substance use and dependency) [9,10,11,12] and behavioral problems (e.g., aggression, criminality) [13,14].

In recent years, researchers have shifted their focus beyond the intrapersonal lens, and instead are beginning to explore the impact of ACEs at an interpersonal and intergenerational level, particularly through parenting behaviors. Studies have demonstrated that mothers with a history of ACEs are often less sensitive, warm and responsive, and instead tend to be emotionally unavailable to their children [15,16]. The evidence has also suggested that ACE-exposed mothers are likely to adopt authoritarian parenting characterized by aggressive and hostile behaviors [17]. Specifically, a maternal history of maltreatment is a predictor of neglectful parenting, and oftentimes, the physical and psychological abuse of offspring [17,18,19]. Studies that have explored this phenomenon with multiple generations have also demonstrated interesting findings. For instance, earlier findings have suggested that marital aggression (G1) predicted antisocial behaviors, alcohol use disorders and intimate partner violence, subsequently leading to aggressive behaviors in G3 [20]. In recent years, Badeness-Ribera and colleagues [21] have demonstrated that physical and psychological abuse (G1) predicted sexual and physical victimization (G2), which was indirectly linked to the physical and sexual victimization of G3, perpetrated by G2. Some researchers have attributed these findings to a diminished maternal self-efficacy stemming from unresolved distress pertaining to past experiences of trauma [22]. These less effective parenting behaviors can contribute to insecure parent–child attachments [23], which research suggests mediates the effects of maternal ACEs on child developmental trajectories [24].

Epidemiological and neurocognitive research has sought to elucidate the psychophysiological pathways between parental ACEs and child development. Scholars have suggested that chronic stress can lead to the repeated activation of the hypothalamic–pituitary–adrenal (HPA) axis, resulting in abnormal cortisol production [25,26]. Over time, this can lead to physiological alterations in the brain [27] that contribute to emotion regulation difficulties in adulthood [28]. Early caregiver interactions, characterized by affective attunement, containment and repair, provide infants with a strong social regulator of HPS axis activity [29] and a template for coherent emotional self-representation [30]. Similarly, parent–child synchrony, the co-regulatory process that shapes interactions through contingent responding and social reciprocity, allows for the coordination of behavioral and biological states [31]. Studies using hyperscanning techniques have shown that greater parent–child neural synchrony during positive interactions can predict a decrease in infant internalizing symptoms [32]. Similarly, studies exploring cortisol synchrony have demonstrated associations with better child socioemotional adjustment under supportive and relaxed states [33]. As such, disturbances in these interactions, such as through maltreatment, provide offspring few opportunities for attunement and synchrony, leaving them vulnerable to emotion dysregulation [24,30]. This can perhaps manifest as hypervigilance, a heightened threat sensitivity and poor inhibitory control [34] and, over time, lead to internalizing (e.g., depression, anxiety) and externalizing problems (e.g., aggression) [35,36].

Taking a different perspective, others are beginning to stress the protective role of Positive Childhood Experiences (PCEs) on negative offspring outcomes, suggesting equal contribution, with ACES, to the foundation of development across the lifespan [37,38]. PCEs pertain to contextual experiences that facilitate healthy development throughout childhood and adolescence, such as positive relationships and a supportive environment, that foster a sense of belonging [39]. For instance, studies have shown promising results for PCEs being able to counteract the deleterious effects of multiple ACE exposures [40,41], demonstrating an inverse dose–response association between PCEs and negative outcomes. More specifically, researchers have suggested that the presence of PCEs can predict better sleep quality [42], improve psychosocial functioning [43] and lower depressive symptoms, perceived stress and loneliness [44]. At an intergenerational level, studies have demonstrated that parents’ PCEs contribute to the use of nurturing parenting attitudes [45] and the promotion of positive coping and parenting self-efficacy [46]. As such, scholars have suggested that, even in the presence of ACEs, children exhibit fewer psychosocial difficulties and increased well-being when more maternal PCEs are present [47,48]. PCEs and other resilience resources (e.g., social support) might ameliorate outcomes through strengthening allostatic regulation—the process of physiological adaptation to stress to ultimately reestablish homeostasis [49]. While sharing promotive and protective effects that support positive developmental trajectories, the conceptual overlap between PCEs and other resilience resources (e.g., social support) might be distinguished through their temporal occurrence. Specifically, PCEs are fixed, retrospective experiences that have been shown to be associated with increased social and emotional support [40], whereas other resilience resources, such as social support, are dynamic constructs, with both constructs predicting overall resilience [50].

To understand the complexity of intergenerational trauma, several models have been developed. For instance, the Conceptual Model of Historical Trauma [51] posits that epigenetic modifications (i.e., changes to gene expression and phenotype, without modifying the DNA sequence) contribute to the relationship between trauma and health outcomes, which are perhaps transmitted to the offspring’s epigenome through intergenerational means (e.g., breast milk composition) [52,53]. On the other hand, the Dimensional Model of Adversity and Psychopathology (DMAP) [54] adopts a multidetermined perspective, providing emotional, cognitive and neurobiological pathways linking adversity with developmental outcomes. The DMAP suggests that both threat (e.g., physical and sexual maltreatment, intimate partner violence, interpersonal violence) and deprivation (e.g., institutionalization, neglect, socioeconomic hardship) uniquely influence neurodevelopment, with the former appearing to affect emotion reactivity and regulation and the latter appearing to affect cognition [54]. However, one model that is particularly relevant to the present review is the Conceptual model of the Intergenerational Transmission of Emotion Dysregulation in Mothers [55]. This model posits that the adverse effects of childhood maltreatment on emotion regulation interfere with parenting behaviors (e.g., emotional climate of the family, parents’ ability to model adaptive coping strategies, parents’ response to negative offspring emotions and the ability to engage in emotional discussions with offspring), subsequently increasing the risk of emotional difficulties in offspring. The model emphasizes that protective factors (e.g., social support, secure attachments with caregiver, healthy relationships in adulthood, engagement in self-care behaviors) can mitigate the deleterious outcomes of childhood maltreatment on emotion regulation and parenting [56]. However, it also acknowledges that contextual and familial hardships (e.g., socioeconomic hardship, single parenthood, low educational attainment) can exacerbate the difficulties with parental emotional socialization practices and, thus, lead to poor developmental outcomes in offspring. While this model focuses on the experiences of mothers, we seek to use this model with all caregivers, including fathers and nonbiological primary caregivers. Considering its relevance and emphasis on protective factors (which the others appear to lack), investigators have selected the Intergenerational Transmission of Emotion Dysregulation Model [55] to structure the results and narrative synthesis of the present review.

Previous reviews have sought to systematically synthesize the literature ascertaining the relationship between cumulative ACEs and health outcomes [4], the justice system [57] and psychopathology [58], while others have explored the intergenerational effects of parental ACEs on offspring [59,60]. To understand resilience, scholars have also examined the associations between PCEs and adult outcomes in both the presence and absence of childhood adversity [50,61]. However, to the researcher’s knowledge, no such reviews have scoped the intergenerational research of parents’ adverse and positive experiences (in childhood and adulthood) and offspring developmental, psychological and behavioral outcomes. It is important to explore these experiences concurrently as childhood stressors are not only predictive of adult adversities (e.g., victimization, psychopathology), but they both influence offspring experiences of trauma [62] and related biopsychosocial outcomes [47,63]. Additionally, the presence or absence of positive experiences has been shown to moderate such relationships [48,64]. Thus, while we aim to only scope and evaluate the current evidence base, our findings could also support the development of future research, eventually highlighting the specific questions that may be addressed by systematic reviews [65]. Overall, the publications following this review could perhaps foster a better understanding of the pathways of intergenerational trauma and facilitate the development (or amendment) of policies that are inclusive and, where appropriate, culturally sensitive.

This scoping review aimed to collate all studies that have assessed the associations between parental adverse and positive experiences (in childhood and/or adulthood) and the developmental, psychological and behavioral outcomes of their offspring. We aimed to describe the nature of this research to date, explicitly examining the methodological designs and geographical locations, while describing the variables that have been tested (adversity protective factor(s) and offspring outcomes) and salient patterns among the reported results. We also aimed to identify what the limitations and gaps in the intergenerational literature are for the associations between parental adverse and positive experiences (in childhood and adulthood) and the developmental, psychological and behavioral outcomes of their offspring.

### Review Questions

1. What is the nature of the intergenerational research on the associations between parental adverse and positive experiences (in childhood and adulthood) and the developmental, psychological and behavioral outcomes of their offspring?
2. What are the limitations and gaps in the intergenerational research on the associations between parental adverse and positive experiences (in childhood and adulthood) and the developmental, psychological and behavioral outcomes of their offspring?

## 2. Materials and Methods

### 2.1. Design

Scoping reviews are a form of knowledge synthesis that employs a systematic and iterative approach to determine the scope and nature of a body of literature, while identifying potential knowledge gaps [66]. Given that the literature on the intergenerational effects of positive childhood and adulthood experiences is in its preliminary stages, and the paucity of systematic intergenerational research, a scoping review was the appropriate methodological approach in this context. This scoping review was pre-registered with the Open Science Framework (OSF) on 21 May 2025 (https://osf.io/prxtq/overview?view_only=1c54f9e0f0834d09ac842953618e20da), where the study protocol and analysis plan are also available.

This scoping review has adhered to the Preferred Reporting Items for Systematic Reviews and Meta-Analyses extension for Scoping Reviews (PRISMA-ScR) statement [67], allowing for the provision of a comprehensive review, transparent and consistent in nature (see Figure S1).

### 2.2. Search Strategy

**Figure 1:**
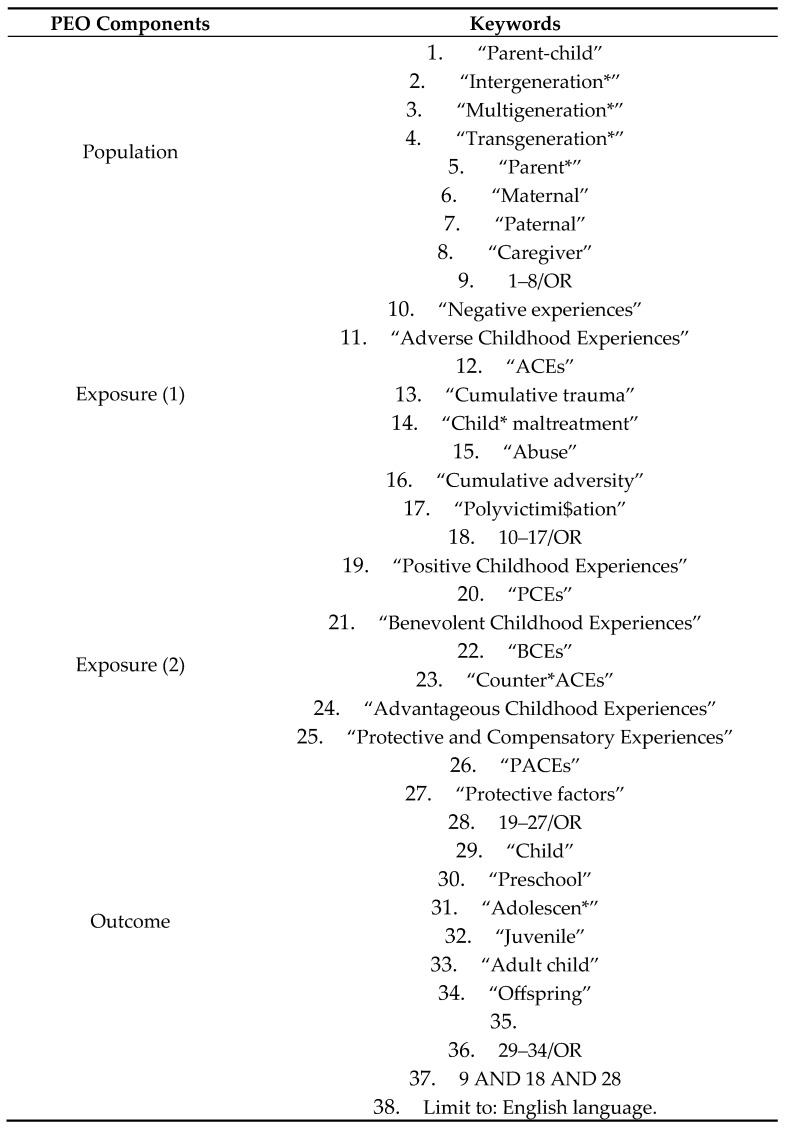
Search Strategy. * represents truncation tool to retrieve results with any ending.

Reviewers developed the inclusion and exclusion criteria consistent with an updated version of the Population, Intervention, Comparator, Outcomes, and Studies (PICOS) framework [68], changing ‘Interventions’ to ‘Exposure’ and omitting ‘Comparator’ (PEOS; see [69] for an example). The search strategy was developed using the keywords of relevant studies, which reviewers adapted to each database: PubMed, Medline (Ovid), Scopus, APA PsycInfo (EBSCOhost), APA PsycArticles (EBSCOhost), Applied Social Sciences Index & Abstracts (ASSIA; ProQuest), Wiley, Web of Science, ProQuest and the Cumulated Index in Nursing and Allied Health Literature (CINAHL; EBSCOhost). Reviewers also conducted a thorough examination of the records that cited included studies and the references cited within the included studies. The full search was conducted on 20 June 2025 and concluded on 27 June 2025. The search strategy is available in Figure 1.

### 2.3. Inclusion/Exclusion Criteria

The population of interest was parent–child dyads, including biological and non-biological (e.g., grandparent, foster parent) primary caregivers. No limits were placed on the age of offspring. We considered records that included parents’ adverse and positive experiences (in childhood and/or adulthood), measured either in singular or cumulative form, and their association with offspring developmental, psychological and behavioral outcomes. We defined parents’ adverse experiences as encompassing all forms of maltreatment including physical, psychological, sexual abuse and neglect (direct and/or vicarious), household dysfunction, exposure to community and/or collective violence; peer or crime victimization; discrimination; and sudden and/or violent bereavements (i.e., accident, homicide, suicide). Similarly, parents’ positive experiences were defined as encompassing positive and supportive relationships; safe family environments and neighborhoods; having beliefs that provided comfort (e.g., spirituality, religion); positive self-image; established routines that included engagement in rewarding daily activities (e.g., employment, volunteering, parenting, school engagement); social engagement opportunities and social belonging; healthy school or working climates; and engagement in regular self-care. Investigators took relevant measures [1,40,70,71,72] into consideration to inform these definitions. We exclusively considered quantitative studies (including dissertations and theses) published or made available in the English language, as the authors were only English-speaking. As we sought to scope the evidence relating to measurable outcomes (e.g., developmental, psychological and behavioral), authors made the decision to exclude qualitative studies. Thus, we expected that included studies would be largely longitudinal or cross-sectional in nature. However, qualitative work could certainly provide valuable insight into intergenerational experiences and resilience processes should scholars wish to replicate this review with a qualitative approach. No restrictions were placed on date of publication as limited results were expected.

### 2.4. Study Selection

**Figure 2:**
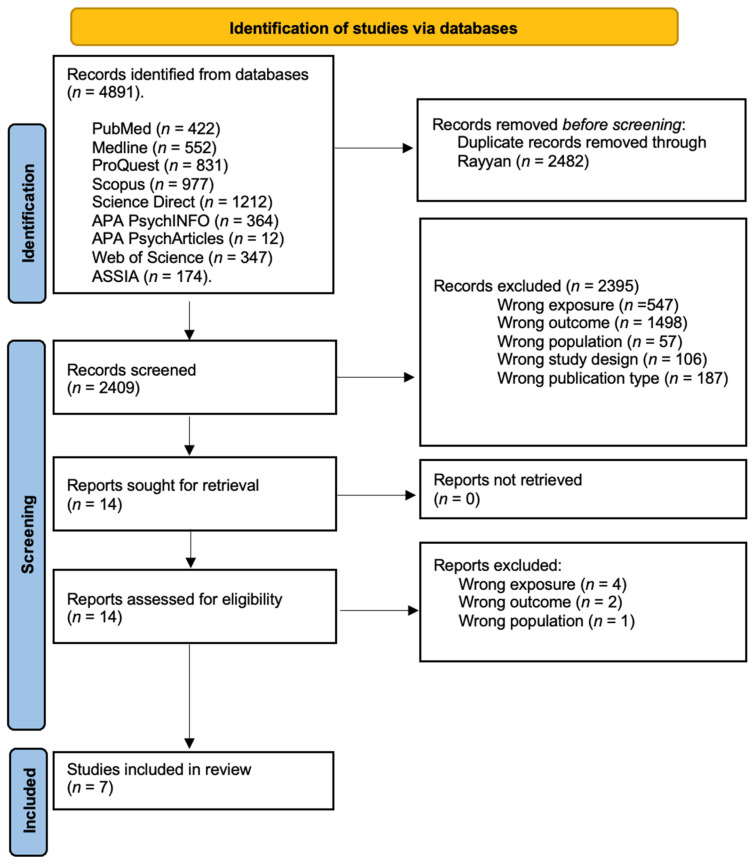
PRISMA Flow Diagram.

The search of ten databases yielded a total of *N* = 4891 articles, which were imported into Rayyan (1.4.3). Following the removal of *n* = 2482 duplicates, *N* = 2409 articles remained for screening. A three-step study selection process was adopted: (1) pilot, (2) title and abstract and (3) full text, conducted by two independent reviewers (MN; CO). Prior to study selection and screening, a two-step (title and abstract, full text) pilot round of 6–8 references was conducted to test and improve (if necessary) the search strategy through Rayyan (1.4.3). Following the completion of the pilot stage (100% agreement), reviewers began the independent two-step screening process, which involved the manual screening of (1) titles, abstracts and keywords and (2) full texts. Interrater reliability for each step was calculated respectively (99.5%, 66.7%). An overview of the reviewed studies, including reasons for exclusion, is illustrated in the PRISMA diagram as shown in Figure 2.

### 2.5. Data Extraction

Two reviewers (MN; CO) independently extracted the following data from each study: (1) first authors name, (2) year of publication, (3) country of publication, (4) study design, (5) sample characteristics (i.e., number of participants, participant age and gender), (6) examined constructs, (7) employed measures and, (8) main findings. Following extraction, both reviewers (MN; CO) presented the data in a descriptive tabular format. To accompany the charted results, the principal investigator (MN) composed a narrative summary structured around the Conceptual Model of the Intergenerational Transmission of Emotional Dysregulation [55]. The summary explored the relationships between and within studies and how they relate to the objectives of the review.

## 3. Results

### 3.1. Selected Studies

Following title and abstract screening, 2395 records were excluded: wrong exposure (*n* = 547), wrong outcome (*N* = 1498), wrong population (*n* = 57), wrong study design (*N* = 106) and wrong publication type (*n* = 187). The full texts of the remaining 14 reports were sought, and a further seven reports were excluded: wrong exposure (*n* = 4), wrong outcome (*n* = 2), wrong study design (*n* = 1) and wrong population (*n* = 1), leaving seven studies for synthesis. Throughout the screening process, screeners were faced with 16 conflicts (title and abstract *n* = 12, full-text *n* = 4) which were discussed to consensus. An updated search was conducted on 27 March 2026; however, no articles met the inclusion criteria.

#### 3.1.1. Study Characteristics

Table 1 provides a summary of the included study’s first author’s name, year of publication, country, design, number of participants, gender distribution and mean age of participants. Table 2 provides a summary of the measures employed and examined constructs. Table 3 provides a summary of study findings. For further details on study findings, see Table S1.

#### 3.1.2. Countries of Data Collection

Concerning country of origin, the majority of the studies took place in the United States (*n* = 6, 85.7%), and the remaining study took place in China (*n* = 1, 14.3%). Notably, no studies addressing the relationship between parents’ adverse and positive experiences and offspring outcomes were found in Central and South Asia, Africa, South America, Australasia, or Europe.

#### 3.1.3. Employed Measures and Examined Constructs

A variety of instruments were used to examine parents’ adverse and positive experiences and offspring outcomes. With respect to parent adversity, cumulative childhood adversity was the most examined construct (*n* = 6, 85.7%), measured most frequently by the Adverse Childhood Experiences Questionnaire (ACE-Q; *n* = 2, 28.6%) [1] along with the international (ACE-IQ; *n* = 1) [87] and Chinese (*n* = 1) derivatives [93]. The original Adverse Childhood Experiences Questionnaire (ACE-Q) [1] contains 10 items, measuring physical and emotional abuse/neglect, sexual abuse and household dysfunction. Similarly, the Adverse Childhood Experiences International Questionnaire (ACE-IQ) [87] expands upon the original ACE questionnaire [1], bringing additional items pertaining to peer, community, and collective violence. Finally, given the sensitive nature and stigma surrounding disclosure in Chinese culture [95,96], the Chinese derivation [93] of the ACE-IQ omits items of sexual abuse. Concerning parents’ positive experiences, cumulative positive childhood experiences (*n* = 4, 57.1%) and social support (*n* = 3, 33.3%) were the most examined constructs. The Benevolent Childhood Experiences (BCEs) [72] scale was the most commonly used instrument (*n* = 2, 28.6%). Finally, with regard to child outcomes, behavioral problems were the most examined construct (*n* = 4, 57.1%), followed by emotional functioning (*n* = 3, 42.9%) and well-being (*n* = 2, 28.6%). All other instruments were only employed in one study, and all other constructs were only examined once.

#### 3.1.4. Adverse Childhood Experiences

Six studies (85.7%) explored the impact of parents’ ACEs. All six studies (*n* = 6, 100%) reported a significant intergenerational association between parent ACEs and negative developmental, psychological and behavioral outcomes in offspring. Notably, one study [47] also showed that parent ACEs had a significant association with child ACEs. That is, an increase of approximately five parent ACEs is expected to increase child ACEs by one. It must be acknowledged that, of the studies that conducted correlations (*n* = 4), weak correlations were reported, ranging from *r* = −0.15 to *r* = 0.33. Overall, weaker correlations were reported in longitudinal research [73] than in cross-sectional research [76,77].

#### 3.1.5. Adult Adversity

Four studies (57.1%) explored adversities in adulthood. The majority of studies (*n* = 3, 75%) reported significant intergenerational associations between various adult adversities (i.e., gendered racial stress, lifetime discriminatory experiences, pregnancy IPV, anxiety and depressive symptoms) and negative developmental, psychological and behavioral outcomes in offspring, with one [76] suggesting male participants to be at particular risk. Notably, Ahmad et al. [73] found that neighborhood violent crime exposure was not associated with negative offspring outcomes. However, they were the only study to explore neighborhood violent crime; therefore, their findings should be approached with caution until further explorations can substantiate their findings. It must also be acknowledged that, of the studies that conducted correlations (*n* = 3), weak correlations were reported, ranging from *r* = 0.13 to *r* = 0.36, with the exception of Hatch et al. [77] for maternal depression to externalizing behaviors (*r* = 0.42). Similar to childhood adversity, weaker correlations were reported in longitudinal research [73] than in cross-sectional research [77].

#### 3.1.6. Positive Childhood Experiences

Four studies (57.1%) explored the buffering effect of cumulative PCEs. Two studies (25%) demonstrated that parent PCEs were both negatively associated with negative offspring outcomes and served as a moderating variable, buffering the relationship between parent adverse and negative psychological outcomes in offspring. That is, as parent PCEs increased, psychopathology outcomes [47] and total difficulties [48] decreased. By contrast, the remaining two studies (25%) suggested that parent PCEs were not related to decreased negative psychological and behavioral outcomes in offspring above the effect of parental adversity. Notably, one study [47] also showed that parent PCEs had a significant association with child PCEs. That is, an increase of approximately three parent PCEs is expected to increase child PCEs by one. It must be acknowledged that one of the two studies that found this relationship to be not significant [74] was the only longitudinal study to explore parent PCEs, with the remaining three studies being cross-sectional in nature. One of the two studies that did find this relationship to be significant [48] had the largest sample size of the data set (*N* = 2586). Thus, further longitudinal explorations are required to ascertain this relationship, especially considering that, of the studies that conducted correlations (*n* = 2), weak correlations were reported (*r* = −0.17 to *r* = 0.13).

#### 3.1.7. Social Support

Three studies (42.9%) explored the buffering effect of social support, with mixed findings. The majority (*n* = 2, 66.7%) of studies found this relationship to be not significant, while the remaining study [77] reported a buffering effect for externalizing behaviors (not internalizing). That is, mothers’ level of exposure to adversity did not influence offspring risk of externalizing behaviors when moderate to high levels of family social support were reported. Further, Ahmad et al. [73] acknowledged that the social-support-by-pregnancy IPV interaction term and social-support-by-SES risk interaction term both demonstrated trend-level patterns, reflecting a potential buffering effect. It is important to note that while Hatch et al. [77] reported a buffering effect, they were the only study of the three to employ a cross-sectional sample design while also having the smallest sample of the data set (*N* = 121). Thus, further longitudinal explorations are required to ascertain this relationship. It must also be acknowledged that, of the studies that conducted correlations (*n* = 2), weak correlations were reported, ranging from *r* = −0.21 to *r* = −0.33. Similar to the adverse childhood and adulthood research, weaker correlations were reported in longitudinal research [73] than in cross-sectional research [77].

#### 3.1.8. Mediators and Moderators

One study (14.3%) conducted mediation analyses to ascertain the pathways between parent ACEs/PCEs and child outcomes. Blackwell et al. [47] explored the mediating role of child ACEs/PCEs, reporting full mediation.

Three studies (42.9%) conducted moderation analyses to ascertain possible variables that affect the strength and direction of the relationship between parent ACEs/PCEs and child outcomes. Various moderating variables were explored, including child sex, maternal knowledge of child development, maternal education, maternal income and social support. All of the aforementioned variables moderated the relationship between parent ACEs/PCEs and child outcomes with the exception of child sex. Notably, while education and income moderated the association between maternal prenatal gendered racial stress and lower child executive functioning, Barbee et al. [75] found this association to be stronger among mothers with higher levels of maternal education and income.

### 3.2. Evaluation of Study Methods

While only seven studies were included in this review, it is important to note limitations. Only three studies included male caregivers (18.16% of total parent sample), and of those studies, female caregivers were the majority. No studies included children in the preadolescent (7–9 years) or adolescent (10–19 years) age groups. All seven studies used self-report measures, most of which (85.71%) were only parent-reported. Finally, only one study [76] conducted a priori power analysis; therefore, findings of this review have likely overestimated true effects.

The majority of studies employed a cross-sectional design (*n* = 4, 57.1%) while the remaining studies were longitudinal (*n* = 3, 42.9%). Two of the longitudinal studies [73,75] collected data in pregnancy at four and three time points, respectively. Specifically, Ahmad et al. [73] collected data during the second and third trimesters of pregnancy, 4 weeks post-birth and 1 year post-birth, while Barbee et al. [75] collected data during the first trimester of pregnancy, during the second or third trimester of pregnancy and two to four years post-birth. The final study [74] utilized data from a nationally representative US survey that collected data biennially from 1968, with eligible respondents having completed the PSID-2013 interview. With regard to population, the majority of the studies included mother–child dyads (*n* = 4, 57.1%), two studies included parent–child dyads (*n* = 2, 28.6%), and one included caregiver–child dyads (*n* = 1, 14.3%). Specifically, the biological mother–biological son dyad was the most popular (*n* = 2483, 35%), closely followed by the biological mother–biological daughter dyad (*n* = 2416, 34.1%). Other less explored dyads included the biological father–biological son (*n* = 177, 2.5%), biological father–biological daughter (*n* = 155, 2.9%), adoptive mother–adoptive daughter (*n* = 1, 0.01%), adoptive mother–adoptive son (*n* = 3, 0.04%), grandmother–granddaughter (*n* = 2, 0.03%), grandfather–granddaughter (*n* = 1, 0.01%) and foster mother–foster daughter (*n* = 1, 0.01%). One study [74] failed to provide the proportions of dyads and has therefore not been included in the aforementioned statistics. The majority of articles (*n* = 6, 85.7%) included children under the age of six. The sample sizes ranged from *n* = 121 to *n* = 2586, with the majority of studies having a sample above *n* = 1000. The majority of parent participants were female (*n* = 6002, 84.6%), followed by 1090 males (15.4%) and four unreported. Parent ages ranged from 25 to 65, with the majority of parents aged between 33 and 35 years. The majority of child participants were female (*n* = 3653, 51.5%), followed by 3443 males (48.5%). Children’s ages ranged from 1 to 38, with the majority of children aged between 3 and 5 years. Notably, no studies investigating the relationship between parents’ adverse and positive experiences and offspring outcomes were found prior to 2021.

## 4. Discussion

The present scoping review sought to collate studies that assessed the associations between parents’ adverse and positive experiences (in childhood and/or adulthood) and the developmental, psychological and behavioral outcomes of their offspring. Specifically, authors described the nature of the studies and identified the limitations and gaps in the intergenerational research to guide further investigations. This review identified only seven articles examining this relationship, demonstrating its infancy. Whilst the studies employed robust methodology, mixed findings were reported, particularly in regard to parents’ positive experiences. Following the narrative synthesis, the following issues were evident: (a) limitations of research samples to date, and (b) limitations of current study designs.

The present review provided insight into the intergenerational associations of parents’ adversity and offspring outcomes. Overall, parents’ exposure to both childhood and adulthood adversity was found to have a direct effect on offspring development (e.g., executive functioning) [75], psychopathology (e.g., internalizing problems) [77] and behavior (e.g., multiple arrests and convictions) [74]. There was one exception reported, which included that of neighborhood violent crime [73]. This finding was unexpected, especially considering that both quantitative [97] and qualitative [98] studies have demonstrated the severe and perpetuating intergenerational threats of neighborhood violence. The authors [73] provided insight into these results, highlighting that the significant association between maternal exposure to neighborhood violent crime and offspring socioemotional–behavioral problems was attenuated once race was included in the fully adjusted model. The authors also acknowledged that the sample largely resided in neighborhoods with less violence (10.4%/1000), demonstrating the complex and perhaps confounding nature of race as a covariate [99]. Further, while direct relationships were reported for adversity and negative child outcomes, the large majority of studies reported weak correlations with the exception of maternal depression-externalizing problems (*r* = 0.42) [77]. Finally, the effect of the relationship between parent adverse and offspring outcomes often differed according to the specific adversity explored [73] or was influenced by moderators (e.g., maternal education) [75] and/or mediators (e.g., child ACEs/PCEs) [47].

Similar to previous literature [100,101], our findings demonstrated that social support moderated the relationship between maternal ACEs and offspring externalizing behaviors. The mechanisms through which social support contributes to offspring development are likely complex and multifactorial, influenced by a number of biopsychosocial factors [102]. For instance, scholars have suggested that social support can buffer against stress, while increasing self-esteem, improving self-regulation and promoting wellbeing [103]. As such, higher perceived social support promotes positive parenting practices [104] through alleviating parenting stress and feelings of isolation [105], thus supporting positive emotional and social development in offspring [106]. Our review further suggested that higher levels of education and income moderated the relationship between prenatal gendered racial stress and lower executive functioning in offspring. While higher educational attainment is often a protective factor against adversity [107], for Black women, this is often met with an increase in exposure to discrimination and gendered racial microaggressions [108]. Thus, perhaps impeding the protective properties of education. Of interest, our review suggested that child sex did not moderate the associations between maternal stressors and offspring socioemotional behavioral problems, contradicting some studies [101,109]. As such, inconsistent findings across the literature might suggest that sex-specific effects are perhaps contingent upon the nature of the prenatal stressor, the developmental stage at which offspring are assessed and the outcomes measured.

While weak to moderate correlations were found for parents’ childhood (*r* = −0.15 to *r* = 0.33) and adulthood (*r* = 0.13 to *r* = 0.42) adversity, our findings are perhaps still practically significant. The intergenerational associations between parents’ experiences and child outcomes are complex and multifaceted, influenced by an array of biological [110], psychological [111] and socioeconomic [112] factors, among others. Therefore, one would not necessarily expect to find large effect sizes from any one single factor. As such, both adverse childhood and adulthood experiences should be considered contributing factors within this multifactorial framework, rather than independent predictors of child outcomes. Of interest, the slightly stronger associations found between adult adversity and child outcomes could suggest that current, dynamic adversities perhaps hold greater practical relevance than those retrospective in nature (i.e., ACEs). For instance, from a clinical perspective, this distinction could suggest that, as opposed to ACEs, adversities in adulthood are more amenable to intervention and could play an important role in the identification of children more susceptible to negative outcomes. Nevertheless, the fairly modest effect sizes demonstrate that neither parents’ adversities in childhood nor adulthood should be considered independently, but rather among other risk and protective factors, thereby informing intervention appropriately.

The present review provided insight into the intergenerational associations of parents’ positive experiences and offspring outcomes. Overall, mixed findings were found for the effect of both childhood (e.g., cumulative PCEs) [76] and adulthood (e.g., social support) [77] positive experiences on offspring development (e.g., executive functioning) [81], psychopathology (e.g., wellbeing and psychopathology) [47] and behavior (e.g., multiple arrests and convictions) [74]. While unexpected, an explanation could be provided for these discrepancies. For instance, of the four that found this relationship to not be significant, two considered caregiver/parent–child dyads [74,76] and one employed the only adult child sample [74]. Further, Barbee et al. [75] were the only study to measure a developmental outcome (i.e., executive functioning), and three of four studies were longitudinal in nature. While the more varied sample and outcome measure (e.g., inclusion of fathers, nonbiological primary caregivers, adult children and developmental outcomes) could suggest more valid and generalizable findings, the studies that found this relationship to be significant had a larger sample (*N* = 4739). Finally, while direct relationships were reported for positive experiences and less negative outcomes, all of the studies reported weak correlations. While one might assume that these findings suggest that parents’ positive experiences are less influential than adversity, it must be remembered that only seven studies were included in this review. Instead, these findings reinforce that the current evidence base is still developing and that further explorations are required. Perhaps scholars might find it beneficial to investigate experiences included/not included (e.g., pet ownership) in the positive childhood/adulthood experience measures and make comparisons between correlations. Further, while weak to moderate correlations were found for parents’ positive childhood experiences (*r* = −0.17 to *r* = 0.13) and social support (*r* = −0.21 to *r* = −0.33), our findings perhaps still have practical significance. Similar to before, parents’ positive experiences in adulthood (i.e., social support) demonstrated more consistent associations than those in childhood, which could perhaps be explained through their temporal occurrence. As such, from a clinical perspective, our findings perhaps suggest that since social support is a modifiable factor, community-based interventions such as peer support groups could play a pivotal role in improving family wellbeing and promoting positive child outcomes.

We also note important limitations of the research samples of included studies. This review found that only three studies included male caregivers *n* = 1090, with the majority being female caregivers *n* = 6002. While the research is preliminary in nature, it is important to highlight this disparity. We emphasize the need to further examine this phenomenon with men, including nonbiological primary caregivers, and populations with different gender identities (e.g., LGBTQ+, non-binary) to advance our understanding of these subgroups. This is particularly relevant given that the aforementioned subgroups are at particular risk of developing varying mental health conditions [113,114] which could perhaps affect parenting behaviors and thus offspring outcomes. The difficulties surrounding male recruitment could perhaps be attributed to hegemonic masculine expectations pertaining to invulnerability, inhibiting men from participating in trauma research through associated feelings of shame [115]. Similarly, authors have suggested that researcher-held biases regarding gender and caregiving are also a significant barrier to male recruitment [116]. Specifically, social norms have perpetuated the premise of fatherhood as an economic provision, while physical and emotional caregiving are perceived as inherently feminine [117]. Of note, the research investigating the underrepresentation of fathers has reported that 80% had merely not been asked to participate [118], demonstrating the negative implications of traditional social norms regarding gender roles and caregiving in the literature. Secondly, this review highlighted that adolescents and adult children remain under-researched in this area, with most of the studies focusing on children under the age of six. Throughout the development period (12–19 years), adolescents are at an increased risk of both trauma exposure and multiple forms of psychopathology [119,120], in addition to the standard hormonal, metabolic, and neural changes of this time [121]. Thus, given these burdens, it is important that future research focuses on the intergenerational impacts of parents’ adverse and positive experiences with this neglected cohort to ensure interventions support this population appropriately. Finally, the authors could not locate any studies beyond the US and China. While it is beneficial that existing literature has examined this phenomenon from an individualist and collectivist perspective, future research must look to expand the literature with different cultural environments to ensure adequate representation and to avoid bias.

We also note important limitations of the designs of included studies. This review found that four of seven studies (57.4%) were cross-sectional. Whilst cross-sectional studies are inexpensive and time efficient [122], they cannot establish temporal relationships between exposures and outcomes, precluding inferences on causality [123]. Further, the self-report nature of the studies assumes that participants will both understand and respond accurately to the measure [124], while the retrospective component is vulnerable to recall biases [125]. This is particularly relevant as retrospective recall of childhood adversity can be affected by several factors including, (a) sleep deprivation, (b) infantile and post-traumatic amnesia, (c) personality and individual differences (e.g., social cognition), (d) source-monitoring errors, and (e) the degree of rehearsal and consolidation of traumatic memory [126,127,128]. Such limitations emphasize the need for longitudinal studies, as this would allow researchers to collect data both prospectively and retrospectively through multiple time points while making comparisons either within or between groups [129]. Future studies would also benefit from using a variety of measurements (i.e., teacher-reported, clinical assessments of offspring outcomes) to minimize bias. Finally, statistical power must be addressed. Four studies [47,48,75,77] failed to provide power calculations or justifications for their sample sizes. Two studies [73,74] did not provide power calculations but suggested their studies were perhaps powered/underpowered, respectively. Finally, while one study [76] did provide power calculations, the authors also acknowledged that they were not adequately powered. The lack of appropriate sample sizes emphasizes the need for further adequately powered research to ensure precise and accurate conclusions are drawn from representative results [130].

We also acknowledge important limitations of the present review. Reviewers only used English search terms and limited papers to those published in English, perhaps contributing to the exclusion of relevant studies published in other languages. Culture can influence the ways in which trauma-related distress is perceived, understood and expressed [131]. Thus, demonstrating the importance of incorporating culture into both research and clinical practice for the development of culturally sensitive interventions that seek to reduce Post-Traumatic Stress Disorder (PTSD) symptomology and promote engagement with services [132]. A further limitation includes the use of a narrative synthesis. The conclusions of a narrative synthesis are subject to interpretation, which perhaps compromises the review’s validity through a lack of bias control [133]. Therefore, scholars may wish to replicate the current review in five years, allowing for the literature to proliferate, and conduct a systematic review and meta-analysis to substantiate the findings of this review.

## 5. Conclusions

The present scoping review sought to collate the evidence that assessed the associations between parental adverse and positive experiences (in childhood and/or adulthood) and the developmental, psychological, and behavioral outcomes of their offspring. The large majority of adults worldwide have reported at least one Adverse Childhood Experience [134] and between three and nine Positive Childhood Experiences [135,136,137,138]. Considering the proliferative nature of ACEs in relation to adult adversities, and the potential buffering effect of PCEs against adversity, it is imperative to better understand their intergenerational effects. Findings of this scoping review demonstrated consistent relations between the developmental, psychological and behavioral effects of parental adversity across generations in both cross-sectional and longitudinal research, supporting the tenets of the Conceptual Model of the Intergenerational Transmission of Emotion Dysregulation [55]. However, less consistent relations were found between parents’ positive experiences and offspring outcomes. Mixed findings along with weak correlations confirm the need for further examinations in this topic area. Specifically, we recommend that researchers explore the intergenerational associations between fathers’ and other, non-biological primary caregivers’ experiences and adolescents and adult child outcomes, particularly those from culturally diverse backgrounds. We also recommend that the studies included in this review are replicated in Central and South Asia, Africa, South America, Australasia, and Europe. As the current evidence base is limited to self-report measures, it would be beneficial if future research utilized a variety of measurements, such as teacher-reported or clinical assessments, to measure child outcomes. We would also recommend researchers use reporting guidelines, such as the Strengthening the Reporting of Observational Studies in Epidemiology (STROBE) checklist [139]. Given the difficulties surrounding male recruitment, it would be beneficial that researchers utilize reporting guidelines to ensure sufficient detail is provided throughout manuscripts. Specifically, this would encourage researchers to provide detail on power calculations, locations and periods of recruitment, numbers of potentially eligible vs. included participants, as well as reasons for non-participation. Doing so would perhaps highlight the specific reasons that impact male recruitment, allowing researchers to overcome recruitment difficulties efficiently. Our findings also highlight the pivotal need for early ACE detection, as well as the importance of recognizing adult adversities as influential risk factors that have intergenerational effects [62,63]. Given these findings, we suggest that initiatives such as ACEs Aware consider assisting practitioners in screening for both childhood and adult adversities to provide more balanced, thorough assessments. This could perhaps improve the identification of children who are more susceptible to negative developmental, psychological and behavioral outcomes, thus enabling practitioners to support families affected by intergenerational trauma more appropriately.

## Figures and Tables

**Table 1:** Study Characteristics.

Author	Year of Publication	Country	Study Design	Sample	Parent Variables	Child Variables
Ahmad et al.	2023 [73]	United States	Longitudinal	*N* = 1127 mother-child dyads.	Maternal age (*m* = 26.6, SD = 5.5).	Age (*m* = 1.1, SD = 0.1). Sex (F = 562, M = 565).
Barnert et al.	2023 [74]	United States	Longitudinal	*N* = 1854 parent-child dyads.	Parent age (*m* = 65.1). Sex (M = 753 F = 1101).	Age (*m* = 38.5, SD = 10.9) Sex (F = 1076, M = 778).
Barbee et al.	2025 [75]	United States	Longitudinal	*N* = 266 mother-child dyads.	Maternal age (*m* = 25.2).	Age (*m* = 2.85, SD = 0.53). Sex (F = 136, M = 130).
Blackwell et al.	2024 [47]	United States	Cross-sectional	*N* = 2032 parent-child dyads.	Parent age (*m* = 35.75, SD = 6.24). Sex (F = 693, M = 323).	Age (*m* = 3.60, SD = 1.41). Sex (F = 505, M = 511).
Gissandaner et al.	2024 [76]	United States	Cross-sectional	*N* = 125 caregiver-child dyads.	Caregiver age (*m* = 33.31, SD = 6.81). Sex (F = 108, M = 14, missing = 3).	Age (*m* = 4.00, SD = 0.78). Sex (F = 67, M =58).
Hatch et al.	2021 [77]	United States	Cross-sectional	*N* = 121 mother-child dyads.	Maternal age (*m* = 29.86, SD = 5.32).	Age (*m* = 4.32, SD = 0.76). Sex (F = 60, M = 61).
Zhu et al.	2023 [48]	China	Cross-sectional	*N* = 2586 mother-child dyads.	Maternal age (*m* = 34.38, SD = 4.89).	Age (*m* = 5.35, SD = 0.86). Sex (F = 1247, M = 1340).

Note. m = mean, SD = standard deviation, F = female, M = male and *N* = sample size.

**Table 2:** Measures and Constructs Explored in Included Studies (*n* = 7).

Reference	Types of Parental Adversity Measured	Measure(s) of Parental Adversity	Types of Positive Experience(s) Measured	Measures of Positive Experience(s)	Types of Child Outcome(s) Measured	Measures of Child Outcome(s)
Ahmad et al. [73].	Cumulative childhood adversity, intimate partner violence, socioeconomic risk, and neighborhood violent crime.	Traumatic Life Events Questionnaire [78]; short-form version of the revised Conflict Tactics Scale [79]; national register of crime.	Maternal social support.	The short-form version of the Social Support Questionnaire [80].	Socioemotional and behavioral problems.	The Brief Infant Toddler Social and Emotional Assessment (BITSEA) [81].
Barnert et al. [74].	Cumulative childhood adversity.	Items on the Panel Study of Income Dynamics of the Childhood Retrospective Circumstances Study (PSID-CRCS) [82] were matched with items from the Adverse Childhood Experiences questionnaire [1].	Cumulative positive childhood experiences.	Items on the Panel Study of Income Dynamics (PSID) for the Childhood Retrospective Circumstances Study (CRCS) [82] were matched with items from the Positive Childhood Experience [40] and Benevolent Childhood Experiences [72] questionnaires.	Criminal legal involvement (child arrest and conviction).	Data collected by the Panel Study of Income Dynamics (PSID) for the Childhood Retrospective Circumstances Study (CRCS) [82].
Barbee et al. [75].	Lifetime experiences of racial discrimination and gendered racial stress.	The Experiences of Racial Discrimination scale [83] and the Jackson, Hogue, Phillips, Contextualised Stress (JHP) scale [84].	Social support.	The coping subscale of the Jackson, Hogue, Phillips Contextualised Stress (JHP) scale [84].	Executive functioning.	The Behaviour Rating Inventory of Executive Functioning-Preschool Version (BRIEF-P) [85].
Blackwell et al. [47].	Cumulative childhood adversity.	Adverse Childhood Experiences questionnaire [1].	Cumulative positive childhood experiences.	The Positive Childhood Experiences Score [40].	Well-being and psychopathology.	Items from the Patient-Reported Outcomes Measurement Information System (PROMIS) [86] Early Childhood norming study.
Gissandaner et al. [76].	Cumulative childhood adversity and anxiety.	Adverse Childhood Experiences-International Questionnaire (ACE-IQ) [87].	Cumulative positive adult and cumulative childhood experiences.	The Adult Resilience Measure-Revised (ARM-R) [88] and the Benevolent Childhood Experiences (BCEs) [72].	Behavioral and emotional functioning.	The Behaviour Assessment System for Children-3 (BASC-3) [89].
Hatch et al. [77].	Cumulative childhood adversity and maternal depressive symptoms.	Adverse Childhood Experiences survey [1] and the Center for Epidemiological Studies Depression Scale Revised (CESD-R) [90].	Perceived family social support.	Multidimensional Scale of Perceived Social Support (MSPSS) [91].	Behavior problems.	Child Behaviour Checklist for ages 1.5–5 [92].
Zhu et al. [48].	Cumulative childhood adversity.	The Chinese version of the Adverse Childhood Experiences International Questionnaire (ACE-IQ) [93].	Cumulative positive childhood experiences.	The Benevolent Childhood Experiences (BCEs) [72].	Psychosocial well-being.	The Chinese version of the Strengths and Difficulties Questionnaire (SDQ) [94].

**Table 3:** Main Findings.

	Main Findings		
Reference	Parent Adversity to Child Outcomes	Parents Positive Experiences to Child Outcomes	Moderators and/or Mediators
Ahmad et al. [73].	Maternal childhood trauma exposure, SES risk, and pregnancy IPV were independently positively associated with socioemotional-behavioral problems. However, neighborhood violent crime was not.	Prenatal social support did not have a significant buffering effect on any association.	Maternal knowledge of infant development moderated associations between both maternal CTE and SES risk on socioemotional-behavioral problems. Child sex was not significant.
Barnert et al. [74].	Four or more parental ACEs was associated with higher odd of arrest and conviction.	Parent PCEs were not related to decreased criminal involvement above the effect of parent ACEs and covariates.	Not applicable.
Barbee et al. [75].	Prenatal reports of gendered racial stress and lifetime discriminatory experiences were significantly associated with executive functioning.	Social support did not have a significant buffering effect on any associations.	Maternal education had a significant moderating effect on the relationship between maternal prenatal gendered racial stress (not discriminatory experiences) and executive functioning problems. Maternal income moderated all associations.
Blackwell et al. [47].	Parent ACEs had a significant association with child ACEs and PCEs. Indirect paths from parents ACEs to child outcomes were only significant for depressive symptoms, curiosity, persistence, positive affect, and frustration tolerance.	Parent PCEs had a significant association with child PCEs. All indirect paths from parent PCEs to child outcomes were significant.	Parent ACEs and PCEs influenced child outcomes via child ACEs/PCEs for all but two outcomes (i.e., anger/irritability, curiosity).
Gissandaner et al. [76].	Caregiver anxiety was positively correlated with externalizing problems, particularly in boys. Caregiver cumulative ACEs demonstrated a moderate significant positive effect on externalizing problems.	Caregiver PCEs were not related to decreased externalizing problems above the effect of caregiver cumulative ACEs and covariates.	Not applicable.
Hatch et al. [77].	There was a positive correlation between maternal ACEs and maternal depressive symptoms and internalizing and externalizing behaviors.	Maternal social support had a buffering effect for the relation between maternal ACEs and externalizing behaviors but not internalizing behaviors.	Maternal social support moderated the association between maternal ACEs and externalizing behaviors but not internalizing behaviors.
Zhu et al. [48].	The offspring of mothers who report high ACEs had an increased likelihood of total difficulties and prosocial problems.	The offspring of mothers who report high PCEs were less likely to be at risk for total difficulties and prosocial problems.	Not applicable.

## Data Availability

No new data were created or analyzed in this study. Data sharing is not applicable to this article.

## Supplementary Materials

The following supporting information can be downloaded at: https://www.mdpi.com/article/10.3390/healthcare14152253/s1, Figure S1: Preferred Reporting Items for Systematic review and Meta-Analyses extension for Scoping Reviews (PRISMA-ScR) Checklist, Table S1: Study Findings with Additional Detail. Table S2: Outcomes Assessed and Found.

## Abbreviations

The following abbreviations are used in this manuscript:

ACEs	Adverse Childhood Experiences
PCEs	Positive Childhood Experiences
BCEs	Benevolent Childhood Experiences
IPV	Intimate Partner Violence
