# Supplementary material for: Exploring the Evidence Base on the Association Between Parents’ Adverse and Positive Experiences and the Developmental, Psychological and Behavioral Outcomes of Their Offspring: A Scoping Review
Source: Healthcare (Basel). 2026 Jul 23;14(15):2253. doi: 10.3390/healthcare14152253 (PMC13464776; doi:10.3390/healthcare14152253)
Supplement: Supplementary file 1 [file healthcare-14-02253-s001.zip › Table S1.pdf]

Table S1

Study Findings with Additional Detail

Reference	Main Findings		
	**Parent adversity to child outcomes**	**Parents positive experiences to child outcomes**	**Moderators and/or mediators**
Ahmad et al. [73].	Maternal childhood trauma exposure (β = 0.38, p = 0.043, 95% CI: 0.01, 0.74), SES risk (β = −1.03, p < 0.001, 95% CI: [−1.58, −0.48]), and pregnancy IPV (β = 0.48, p = 0.012, 95% CI: [0.11, 0.86]) were significantly associated with childhood problems, however, neighbourhood violent crime was not (β = 0.35, p = 0.11, 95% CI: [−0.08, 0.79]).	Prenatal social support did not have a significant buffering effect on any association between CTE, SES risk, pregnancy IPV or neighbourhood violent crime. However, the social-support-by-pregnancy IPV interaction term and social support-by-SES risk interaction term both demonstrated trend-level patterns ($\beta_{interaction}$ = −0.35, p = 0.07, 95% CI: [−0.73, 0.03]; $\beta_{interaction}$ = 0.38, p = 0.09, 95% CI: [−0.06, 0.83]), respectively, reflecting a potential buffering effect.	Maternal knowledge of infant development moderated the association between maternal SES risk ($\beta_{interaction}$ = 0.60, p = 0.020, 95% CI: [0.09–1.10]) and maternal CTE ($\beta_{interaction}$= 0.43, p = 0.042, 95% CI: [0.02–0.85]) on child socioemotional-behavioural problems. Moderation tests by child sex were not significant.
Barnert et al. [74].	Offspring of parents who reported four or more ACEs had higher odds of arrest before age 26 and conviction, both before (aOR, 1.91; 95% CI: [1.14-3.22]; aOR, 3.22; 95% CI: [1.62-6.40]) and after (aOR, 1.78; 95% CI, 1.04-3.03; aOR, 3.01; 95% CI: [1.53-5.93]) controlling for	Offspring of parents who reported four or five PCEs had a lower risk of multiple arrests vs no arrests before controlling for ACEs (adjusted RRR, 0.41; 95% CI: [0.22-0.77]). However, after incorporating ACEs, this association was no longer significant (adjusted RRR, 0.53; 95% CI: [0.28-	Not applicable.

	parental PCEs and covariates, respectively. Similarly, offspring of parents who reported four or more ACEs were at higher risk of multiple arrest versus no arrest (adjusted RRR, 3.93; 95% CI: [1.94-7.93]), one conviction versus no convictions (adjusted RRR, 2.57; 95% CI: [1.21-5.42]) or multiple convictions vs no convictions (adjusted RRR, 4.90; 95% CI: [1.38-17.32]), adjusting for parental PCEs and covariates, respectively.	1.00]). Offspring of parents who reported four or five PCEs had lower odds of conviction compared to those with 0-1 PCEs (aOR, 0.53; 95% CI: [0.29-0.99]) before controlling for ACEs. However, after incorporating ACEs, this association was no longer significant (aOR, 0.65; 95% CI: [0.35-1.19]).	
Barbee et al [75].	Maternal prenatal reports of gendered racial stress were significantly associated with executive functioning; inhibitory self-control (β = .165, p = .006, 95% CI: [.172, 1.020]), flexibility (β = .167, p = .005, 95% CI: [.135, .769]) and emergent metacognition (β = .126, p = .039, 95% CI: [.024, .912]). All subscales were significantly associated with gendered racial stress with the exception of working memory (β = .106, p = .084, 95% CI: [-.034, .530]). Maternal prenatal reports of lifetime discriminatory experiences were significantly associated	Social support did not have a significant buffering effect on any associations between maternal racial discrimination or gendered racial stress and offspring executive functioning, respectively, inhibitory self-control (t = -.842, p = .401, CI: -[.066, 027]; t = .863, p = .389, CI: [-.003, 008]), flexibility (t = .055, p = .956, CI: [-.034, 036]; t = 1.262, p = .208, CI: [-.002, 007]) and emergent metacognition (t = -.549, p = .584, CI: [-.062, 035]; t = .296, p = .767, [CI: -.005, 007]).	Maternal education had a significant moderating effect on the relationship between maternal prenatal gendered racial stress (not discriminatory experiences) and executive functioning problems; inhibitory self-control (R^2 = .110, p = .036, 95% CI [.004, 105], flexibility (R^2 = .104, p = .042, 95% CI [.001, 078] and emergent metacognition.<br><br>Maternal income moderated the relationship between maternal prenatal gendered racial stress and

	with executive functioning; inhibitory self-control (β = .201, $p <$ .001, 95% CI: [.038, .141]), flexibility (β = .200, $p <$ .001, 95% CI: [.028, .105]) and emergent metacognition (β = .162, p = .008, 95% CI: [.020, .128]).		discriminatory experiences and executive functioning problems.
Blackwell et al. [47]	Parent ACEs had a significant association with child ACEs and PCEs. That is, each additional parent ACE equated to 0.18 (95%CI: [0.06, 0.31]) increase in child PCEs and 0.13 (95% CI: [0.08, 0.18) increase in child ACEs. In turn, each additional child ACE equated to 0.10-0.18 SD increase in psychopathology.<br><br>*Indirect paths from parents ACEs to child outcomes were only significant for depressive symptoms ($\hat{b}$ = -0.03, 95% CI [-0.0, 0.01], curiosity ($\hat{b}$ = 0.03, 95% CI [0.00, 0.05], persistence ($\hat{b}$ = 0.05, 95% CI [0.02, 0.08], positive*	Parent PCEs had a significant association with child PCEs. That is, each additional parent PCE equated to 0.34 (95% CI: [0.20, 0.45) increase in child PCEs. In turn, an increase in 1 child PCE was related to a 0.10-0.16 SD increase in well-being and a 0.06-0.10 decrease in psychopathology.<br><br>*All indirect paths from parent PCEs to child outcomes were significant; positive affect ($\hat{b}$ = 0.05, 95% CI [0.03, 0.08]), curiosity ($\hat{b}$ = .005, 95% CI [0.03, 0.07]), persistence ($\hat{b}$ = 0.06, 95% CI [0.03, 0.08]), flexibility ($\hat{b}$ = 0.06, 95% CI [0.03, 0.09]), frustration tolerance ($\hat{b}$ = 0.04, 95% CI [0.02,*	*Parent ACEs and PCEs influenced child outcomes via child ACEs/PCEs for all but two outcomes (i.e., anger/irritability, curiosity); positive affect ($\hat{b}$ = -0.01, 95% CI [-0.02, -0.00], persistence ($\hat{b}$ = -0.01, 95% CI [-0.01, -0.00], flexibility ($\hat{b}$ = -0.01, 95% CI [-0.02, -0.00], anxiety ($\hat{b}$ = 0.01, 95% CI [-0.00, 0.01] and depressive symptoms ($\hat{b}$ = 0.01, 95% CI [0.00, 0.01].*

	affect ($\hat{b}$ *= -0.01, 95% CI [-0.02, 0.07], and frustration tolerance (*$\hat{b}$ *= 0.03, 95% CI [0.01, 0.06].*	*0.06]), anxiety (*$\hat{b}$ *= -0.03, 95% CI [-0.05, -0.01]), depressive symptoms (*$\hat{b}$ *= -0.04, 95% CI [-0.06, -0.02] and anger/irritability (*$\hat{b}$ *= -0.02, 95% CI [-0.04, -0.01]).*	
Gissandaner et al. [76].	Caregiver anxiety was positively correlated with preschool externalising problems (β *= 0.39, 95% CI [-0.74, -0.05]*), with boys demonstrating greater externalising problems than girls. Caregiver cumulative ACEs demonstrated a moderate (β = - 0.22, p = < 0.05, 95% CI [0.04, 0.40) significant positive effect on offspring externalising problems.	Caregiver PCEs were not related to decreased offspring externalising problems above the effect of caregiver cumulative ACEs and covariates (β = -0.12, p = 0.20, 95% CI [-0.29, 0.06]).	Not applicable.
Hatch et al. [77].	There was a positive correlation between maternal ACEs and maternal depressive symptoms and offspring internalising (r = .23, p < .05; r = .23, p < .05) and	Maternal social support had a buffering effect for the relation between maternal ACEs and offspring externalising behaviours *(*β *= -.32, p = .01, 95% CI [-0.24, -0.05]) but*	Maternal social support moderated the association between maternal ACEs and offspring externalising

	externalising behaviours (*r* = .33, *p* < .01; *r* = .42, *p* < .001), respectively.	*not internalising behaviours (β = -.18, p = .06, 95% CI [-0.12, 0.00]).*	behaviours (*b* = -.14, *p* <.01), but not internalising behaviours (*b* = -.06, *p* = .06).
Zhu et al. [48].	The offspring of mothers who report high ACEs had an increased likelihood of total difficulties (OR = 1.18; 95%CI = [1.13, 1.27]) and prosocial problems (OR = 1.07; 95%CI = [1.02, 1.14]).	The offspring of mothers who report high PCEs were less likely to be at risk for total difficulties (OR = 0.88; 95%CI = [0.83, 0.94]) and prosocial problems (OR = 0.93; 95%CI = [0.87, 0.99]), even after incorporating for ACEs and covariates.	Not applicable.
