# Supplementary material for: Exploring the Evidence Base on the Association Between Parents’ Adverse and Positive Experiences and the Developmental, Psychological and Behavioral Outcomes of Their Offspring: A Scoping Review
Source: Healthcare (Basel). 2026 Jul 23;14(15):2253. doi: 10.3390/healthcare14152253 (PMC13464776; doi:10.3390/healthcare14152253)
Supplement: Supplementary file 1 [file healthcare-14-02253-s001.zip › Table S2.pdf]

Table S2

Outcomes assessed and found

Author	Outcomes Assessed	Outcomes Found
Ahmad et al. [73]	*ACEs*: Physical abuse, sexual abuse, IPV exposure. *AAEs*: experienced IPV, neighbourhood violent crime. *PAEs*: Maternal social support *Child outcomes*: Socioemotional and behavioural problems including, internalising, externalising and dysregulation problems.	~ 36% of women reported at least one CTE, 67.2% reported one form of IPV during pregnancy and the mean neighbourhood violent crime rate was 13.9%/1000 residents. The mean score for child outcomes was m = 9.7 with 25% falling in the behavioural issue range.
Barnert et al. [74]	*ACEs*: Physical abuse, emotional abuse, sexual abuse or assault, emotional neglect, IPV exposure, household dysfunction. *PCEs*: Peer support and healthy school climate, neighbourhood safety and support, nurturing parental relationship. *Child outcomes*: Adult child arrest and conviction.	~ 32.4% of parents reported at least one CTE and 50.2% had between two and three PCEs. Of adult children with a parent that had at least one ACE, 17.5% had been arrested before age 26.
Barbee et al. [75]	*AAEs*: Discrimination and gendered racial stress. *PAEs*: Social support. *Child outcomes*: Executive functioning.	Mean discrimination (*m* = 2.21, *SD* = 2.40), pregnancy discrimination (*m* = 96.48, *SD* = 19.57), social support (*m* = 32.94, *SD* = 9.12). Racial stress (*missing*), executive functioning (*missing*).
Blackwell et al. [47]	*ACEs*: Emotional, physical and sexual abuse, IPV exposure, emotional and physical neglect, household dysfunction. *PCEs*: Positive experiences relating to family, friends and the community. *Child outcomes*: Positive	~ 19.69% of parents reported at least one CTE and 30.80% reported between 0 and 2 PCEs. ~ 10.84% of children had at least one emotional behavioural or developmental delay or disorder.

	affect, curiosity, persistence, flexibility, frustration tolerance, anxiety, depressive symptoms, and anger/irritability.	
Gissandaner et al. [76]	*ACEs*: Emotional and physical neglect, emotional, physical and sexual abuse, household dysfunction, bullying and community and collective violence. AAEs: Anxiety, depressive and PTSD. *Resilience factors*: Supportive friends and family, access to adequate food, opportunities for employment and problem-solving, *PCEs*: Relational and internal safety and security, positive predictive quality of life and interpersonal support. *Child outcomes*: Externalising problems.	Mean ACE score (*m* = 7.23, *SD* = 4.97), resilience factors (*m* = 57.35, *SD* = 10.45), PCEs (*m* = 8.90, *SD* = 1.78), externalising problems (*m* = 17.00, *SD* = 7.80).<br>Caregiver ADHD = 8, anxiety = 35, bipolar disorder = 6, BPD = 2, depression = 38, oppositional defiant disorder = 1, PTSD = 13, substance use disorder = 1 and other = 6.
Hatch et al. [77]	*ACEs*: Physical and emotional abuse and neglect, sexual abuse and household dysfunction. *AAEs*: Depressive symptoms. *PAEs*: Social Support. *Child outcomes*: Externalising and internalising behaviours.	Mean ACE score (*m* = 3.08, *SD* = 2.46), depression (*m* = 0.50, *SD* = 0.53), social support ranging from 4-28 (*m* = 20.81, *SD* = 7.71), internalising (*m* = 7.31, *SD* = 6.23) and externalising behaviours (*m* = 9.80, *SD* = 8.54).
Zhu et al. [48]	*ACEs*: Emotional abuse and neglect, physical abuse and neglect, peer bullying, community violence and household dysfunction. *PCEs*: Predictable quality of life, social support, internal and relational safety. *Child outcomes*: Conduct problems, hyperactivity, emotional problems, peer problems and prosocial behaviour.	Mean ACE score (*m* = 3.44, *SD* = 1.85) and PCEs score (*m* = 9.10, *SD* = 1.45). Children at risk of total difficulties 19.7% and prosocial problems 21.3%.

Note: ACEs = Adverse Childhood Experiences, AAEs = Adverse Adult Experiences, PCEs = Positive Childhood Experiences, PAEs = Positive Adult Experiences, IPV = Intimate Partner Violence, CTE = Childhood Trauma Exposure, PTSD = Post-traumatic Stress Disorder, ADHD = Attention Deficit/Hyperactivity Disorder, BPD = Borderline Personality disorder,
